# Antidote to Arsenic

**Published:** 1851-07

**Authors:** 


*Antidote to Arsenic.*—We publish the following as a fact which savors of great usefulness, from the British American Med. and Surg. Journal.

"Mr. Lucas, of Beauvais, states, that in as many as nine cases of poisoning with arsenic, he has found calcined magnesia arrest the symptoms of poisoning, and remove its effects." An antidote so common is a valuable discovery.

